# Mechanism of Tailoring Laser-Induced Periodic Surface Structures on 4H-SiC Crystal Using Ultrashort-Pulse Laser

**DOI:** 10.3390/nano15181398

**Published:** 2025-09-11

**Authors:** Erxi Wang, Chong Shan, Xiaohui Zhao, Huamin Kou, Qinghui Wu, Dapeng Jiang, Xing Peng, Penghao Xu, Zhan Sui, Yanqi Gao

**Affiliations:** 1 Shanghai Institute of Laser Plasma, China Academy of Engineering Physics, 1129 Chenjiashan Road, Shanghai 201800, China; erxi_wang@163.com (E.W.); xhzhao_silp@163.com (X.Z.); lqling@vip.163.com (Z.S.); yqgao_silp@163.com (Y.G.); 2 State Key Laboratory of Functional Crystals and Devices, Shanghai Institute of Ceramics, Chinese Academy of Sciences, Shanghai 201899, China; huaminkou@mail.sic.ac.cn (H.K.); wuqinghui@mail.sic.ac.cn (Q.W.); dajiang@mail.sic.ac.cn (D.J.); 3 College of Intelligent Science and Technology, National University of Defense Technology, Changsha 410073, China; pengxing22@nudt.edu.cn; 4 Troops 91007, Chinese People’s Liberation Army; zhenqing20072008@sina.com

**Keywords:** laser-induced periodic surface structures, 4H-SiC crystals, femtosecond–picosecond lasers, photothermal weak absorption

## Abstract

In this study, we examine the characteristics of laser-induced periodic surface structures (LIPSSs) fabricated on N-doped 4H-SiC (N-SiC) and high-purity 4H-SiC (HP-SiC) crystals using femtosecond–picosecond lasers. The effects of various laser parameters on the orientation, size, and morphology of the LIPSS are systematically investigated. The results reveal that, under identical laser irradiation conditions, the area of LIPSS on both N-SiC and HP-SiC increases linearly with the number of pulses, with N-SiC exhibiting a higher growth coefficient. Furthermore, analysis of differences in photothermal weak absorption and electric field modulation during the LIPSS fabrication process indicates that distinct SiC crystals yield varied LIPSS formation outcomes. This work not only elucidates the underlying physical mechanisms governing LIPSS formation on different silicon carbide crystal surfaces but also provides valuable guidance for precisely controlling the size and orientation of LIPSS regions on various 4H-SiC substrates.

## 1. Introduction

Silicon carbide (SiC) is an exceptional wide-bandgap semiconductor, distinguished by its high bandgap [1,2,3], excellent thermal conductivity [4,5], robust breakdown electric field strength [6], and substantial electron mobility [7]. It plays an irreplaceable role in critical fields such as efficient energy conversion in the new energy sector [8], high-temperature electronics in aerospace applications [9], fusion diagnostics [10], neutron detection [11], luminescent devices [12], and high-frequency microwave devices in the electronics and information industry [13,14]. With continuous advancements in silicon carbide crystal application technology—particularly in the development of third-generation semiconductor devices, novel optoelectronic devices, and micro–nano-electromechanical systems—the surface properties of SiC are increasingly required to be refined and functionalized [15,16,17]. In this context, advanced micro–nano-processing techniques have become key to overcoming traditional application challenges and exploring new scenarios, enabling the construction of micro–nano-structures with tailored geometries, size distributions, and functional characteristics on the SiC surface. For example, Lu et al. employed nanosecond laser etching to create various micro-textures on the SiC surface, which, when combined with chemical modification, impart hydrophobic drainage properties [18]. These micro- and nanostructures not only significantly alter the optical, electrical, mechanical [19], and chemical properties of material surfaces but also promote special functionalities such as enhanced light absorption efficiency [20], optimized electron transport pathways [21], and improved surface catalytic activity [22]. Collectively, these advances provide critical technological support for enhancing the performance of SiC-based optoelectronic devices, developing novel sensor technologies, and expanding applications in energy catalysis.

SiC crystals, a prototypical hard and brittle material, can be processed more efficiently via laser-induced periodic surface structures (LIPSSs) compared to other laser processing techniques for fabricating periodic nanoscale features [23,24]. Extensive research has characterized the fabrication properties of LIPSSs. For example, Tomita et al. [25] demonstrated that introducing artificial scratches on the surface of 4H-SiC significantly reduces the energy density threshold for generating high-spatial-frequency LIPSSs (HSFLs), while the threshold for low-spatial-frequency LIPSSs (LSFLs) remains unaffected. Similarly, Yamaguchi et al. [26] reported that in single-crystal 4H-SiC, HSFLs form at lower single-pulse energies, whereas LSFLs originate from the central region and becomes more pronounced as the single-pulse energy increases. Additionally, Miyagawa et al. [27] employed transmission electron microscopy to examine the crystalline state of femtosecond LIPSSs and observed that the resulting micro-nanostructures are produced by periodic etching rather than atomic rearrangement. Yan et al. [28] proposed a LIPSS period prediction model based on the Drude–Sipe model, including free carrier density, and this model successfully predicted the appropriate laser energy density required for the formation of LIPSSs in the experiment, with the calculated LIPSS period very closely matching the experimental results. Zhang et al. [29] found that when preparing LIPSSs, HSFLs could transform into LSFLs once the fluence exceeded the ablation threshold, with the structure array arranged along the main energy flow direction of the linearly polarized scanning laser. Jiao et al. [30] utilized a collinear dual-beam incidence to prepare LIPSSs and studied the regulation of the pulse delay time and polarization angle on the LSFL period. However, systematic studies comparing LIPSS characteristics and underlying physical mechanisms across various SiC crystal types remain absent, a gap that is critical for the precise control of LIPSSs and the broader application of SiC.

In this paper, we investigate precision control techniques for LIPSSs in various types of 4H-SiC materials, along with an analysis of the underlying mechanisms. Experimental studies were conducted to examine the influence of incident energy, pulse durations, polarization states, and pulse counts on the formation of micro–nano-structures on the surfaces of high-purity 4H-SiC (HP-SiC) and nitrogen-doped 4H-SiC (N-SiC). The results reveal dynamic changes in the LIPSS area and provide insight into the conditions and growth characteristics for LIPSS formation on different SiC surfaces under various laser processing parameters. Additionally, photothermal weak absorption testing methods were employed to analyze the weak absorption characteristics of the initial micro–nano-structures on the silicon carbide surfaces. Coupled with simulations of the electric field distribution of the micro–nano-structures on different SiC surfaces, the physical mechanisms governing the formation of LIPSSs induced by various laser parameters were elucidated. This study not only clarifies the influence and mechanisms of ultrashort-pulse laser parameters and silicon carbide material properties on LIPSS formation but also lays the groundwork for achieving precise control of LIPSS areas on different types of SiC crystals.

## 2. Materials and Methods

**Figure 1:**
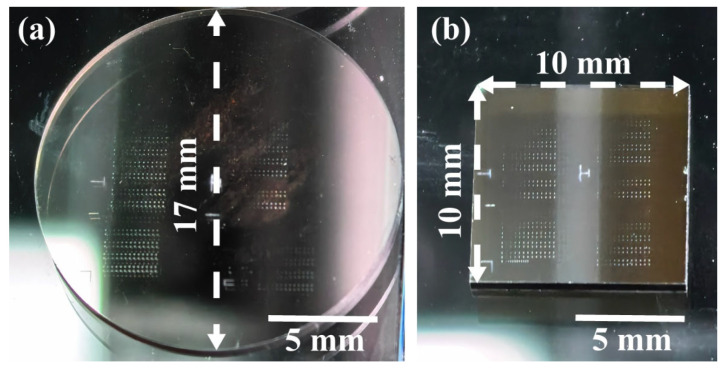
Preparation of micro–nano-structures on the C-face of silicon carbide crystals using ultra-short-pulse lasers (**a**) HP-SiC; (**b**) N-SiC.

In this study, HP-SiC and N-SiC were selected as subjects for investigation, as shown in Figure 1. The surfaces of both SiC crystals were processed using precision polishing technology, ensuring that the surface roughness of each was less than 0.1 nm and met wafer-level standards. The samples were then sectioned into specimens measuring ϕ17 mm × 1 mm and 10 mm × 10 mm × 1 mm, respectively: these two forms of SiC crystals serve distinct functions. High-purity 4H-SiC exhibits superior thermal conductivity, hardness, and stability, rendering it ideal for applications such as mirror substrates and crystal heat sinks; accordingly, it is fabricated into circular shapes using water-guided laser cutting. In contrast, doped 4H-SiC, which possesses electrical conductivity, is predominantly utilized in semiconductor chip production and is thus shaped into squares via grinding wheels. Therefore, the dimensions of two SiC experimental samples were not identical. To objectively evaluate the process and mechanism differences in preparing micro-nano structures on the surfaces of different SiC crystals, the laser irradiation experiments were all conducted on the C-faces of HP-SiC and N-SiC [31].

**Figure 2:**
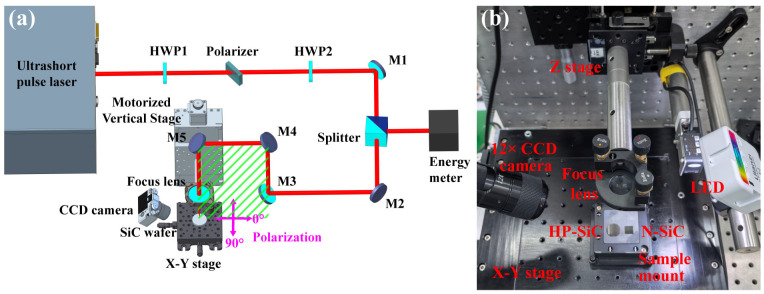
Experiment setup for LIPSS on 4H-SiC. (**a**) Scheme of setup, (**b**) photograph of processing system. HWP, half-wave plate; M, mirror.

This study employs a femtosecond–picosecond pulsed laser with a central wavelength of 1030 nm to irradiate the surface of silicon carbide crystals. The pulse durations are set to 200 fs, 600 fs, 1 ps, and 1.7 ps, respectively, with the system operating at a repetition rate of 200 kHz (Yacto Fiber-FL series custom edition, Yacto Technology, Hangzhou, China). The laser processing apparatus is depicted in Figure 2. Following emission, the ultrashort-pulse laser beams pass through an attenuator, comprising a half-wave plate (HWP1) and a polarizer, which precisely regulates the incident laser energy. Subsequently, an additional half-wave plate is introduced to adjust the polarization of the laser. The beam then traverses a sampling mirror, where the energy of the sampled beam is measured to enable monitoring of the processing energy. Four mirrors are employed to modify the height of the incident laser, and finally, a lens with a focal length of 100 mm focuses the beam to yield a spot radius of 20 μm (1/e^2^). It is worth noting that the incident laser beam is reflected by mirror M5 to impinge on the sample at an angle that is nearly perpendicular. To prevent the beam from retracing its path back to the source, the incident angle is offset by a slight deviation of 1–2° within the plane defined by mirrors M3, M4, and M5. In this plane, the polarization direction perpendicular to it is designated as 90°, while the polarization direction parallel to the plane is defined as 0° (as shown in Figure 2a). The polarization state of the incident beam is adjustable via rotation of the HWP2. By adjusting the z-axis displacement platform mounted on the lens, the ultrashort-pulse laser is accurately aligned to irradiate the surfaces of both HP-SiC and N-SiC. A CCD camera with 12× magnification, positioned on the sample surface, facilitates real-time observation of the laser processing effects, while an x-y-axis displacement platform adjusts the sample’s position relative to the laser beam. These experiments were conducted in ambient air at a temperature of 20 ℃ and a relative humidity of 60% RH. The incident single-pulse energies were set to 6.4 μJ, 12.8 μJ, 19.2 μJ, 25.6 μJ, and 32.0 μJ, respectively, corresponding to the fluence of 0.509 J/cm^2^, 1.019 J/cm^2^, 1.528 J/cm^2^, 2.037 J/cm^2^, and 2.546 J/cm^2^. In this study, four pulse durations were selected for laser irradiation, and their corresponding light intensities were determined through the calculation I=E/S/τ, where E, S, and τ represent the incident laser energy, beam area, and pulsed duration, respectively. (For instance, with an incident laser energy of 19.2 μJ, a spot radius of 20 μm, and pulse duration of 200 fs, 600 fs, 1 ps, and 1.7 ps, the corresponding peak intensities are 7.64 × 10^12^ W/cm^2^, 2.55 × 10^12^ W/cm^2^, 1.53 × 10^12^ W/cm^2^, and 8.99 × 10^11^ W/cm^2^, respectively.) It should be noted that the laser selected for this study operates at a repetition rate of 200 kHz. However, during the preparation of LSFL, the process involves irradiating the sample with a single laser pulse, observing its state using a CCD camera, and then subjecting it to subsequent laser pulses. This sequence is repeated cyclically. Thus, the irradiation is induced by single pulses separated by extended time intervals, resulting in minimal thermal accumulation effects.

This study explores the effects of varying single-pulse energies and pulse counts on the formation of micro- and nano-scale structures. Specifically, different single-pulse laser energies are applied to both HP-SiC and N-SiC samples using multi-pulse sequences ranging from one to twelve pulses. Scanning electron microscopy (SEM) is employed to observe the morphological variations in the laser-irradiated regions, and the influence of pulse energy and pulse number on the surface morphology of silicon carbide is systematically analyzed. Subsequently, an experimental analysis was conducted to investigate the influence of laser pulse duration on the induction of micro–nano-structures. An ultrashort-pulse laser, with pulse durations ranging from 200 fs to 1.7 ps, was employed to fabricate laser-induced periodic surface structures (LIPSSs) via multi-pulse irradiation. This study elucidated the underlying mechanism by which pulse duration modulates the preparation characteristics of micro-nano-structures on various silicon carbide surfaces. Furthermore, by adjusting the half-wave plate angle, lasers with differing polarization states were utilized to generate distinct micro-nano-morphologies, thereby enabling a comprehensive analysis of the associated physical mechanisms.

## 3. Experimental Results

### 3.1. Morphology of LIPSS on the SiC Surface

**Figure 3:**
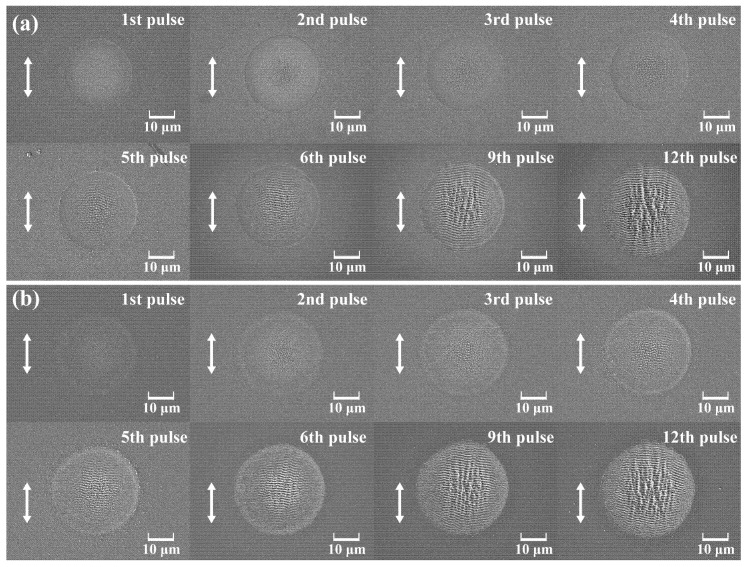
The SEM images of the LIPSS preparation process under the same incident laser parameters (incident laser energy: 19.2 μJ; pulse duration: 200 fs; polarization angle: 90°): (**a**) HP-SiC; (**b**) N-SiC. White arrow shows the electric field vector of the incident laser.

Firstly, we examined the morphology of LIPSSs on various silicon carbide substrates. Using constant laser parameters (incident energy: 19.2 μJ; pulse duration: 200 fs; polarization: S), the LIPSSs on the surface of HP-SiC and N-SiC crystals were prepared. And the morphological evolution during the process was characterized via SEM (TESCAN, Brno, Czech Republic), as illustrated in Figure 3. It could be observed that during multi-pulse laser irradiation, HP-SiC and N-SiC gradually develop scattered micro-undulations in the spot irradiation area. The size of the surface ripple undulations in the laser center area (Gaussian beam peak energy) is larger, while the size of the surface ripple undulations in the spot periphery (lower energy area) is smaller. Under subsequent pulsed laser irradiation, these undulating structures systematically increase in size and depth, eventually forming continuous micro-nano-structures. Larger individual fine ripples in the central region become directly connected, yielding LSFLs with a period of approximately 1 μm, which corresponds to the wavelength of the incident laser. In contrast, the smaller individual fine ripples in the outer region initially connect to form HSFLs with a period ranging from approximately 260 nm to 320 nm; with continued irradiation, these HSFLs subsequently integrate with the central LSFLs, ultimately producing continuous ripples that maintain the same periodicity. Furthermore, the area occupied by these continuous ripple structures gradually increases during successive pulsed laser irradiations, ultimately ceasing to expand once it reaches the ablation zone boundary. Moreover, as the number of laser irradiations increases, the LSFLs in the central region begin to exhibit melting phenomena, forming a collapsed melted structure-oriented perpendicular to the continuous wave patterns and disrupting the continuity of the micro–nano-structures. Therefore, precise fabrication of complete and continuous micro–nano-structures on the SiC surface necessitates strict control over the number of laser irradiations. It is worth noting that during the experiments, following each laser pulse, residual surface material was removed using a nitrogen gas stream. The SEM images indicate that the debris was primarily ejected and deposited outside the ablation zone, thereby exerting a negligible effect on subsequent pulses.

**Figure 4:**
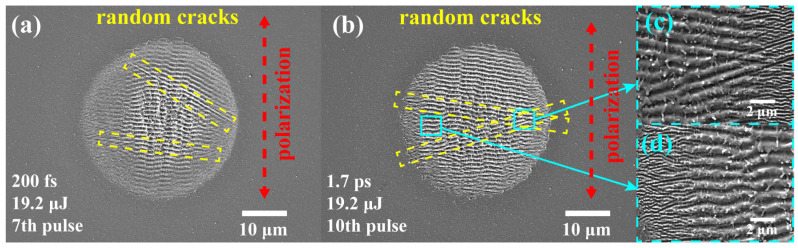
SEM results of LIPSSs prepared on N-SiC surfaces under different incident laser parameter conditions: (**a**) pulse duration 200 fs, energy 19.2 μJ shot number: 7; (**b**) pulse duration 1.7 ps, energy 19.2 μJ shot number: 10; (**c**) high-resolution SEM of crack-affected LIPSSs in (**b**), and (**d**) high-resolution SEM of ideal LIPSS in (**b**).

The experimental results indicate that under identical conditions of single-pulse energy, pulse duration, and polarization, N-SiC (4th shot) exhibits LSFL formation earlier than HP-SiC (5th shot). Moreover, under the same number of pulses, N-SiC produces a larger LSFL area prior to the full formation of LIPSS within the irradiated spot. During the fabrication of the LIPSS on N-SiC surfaces, random cracks are observed within the laser irradiation region. This phenomenon primarily arises from the doping process, which disrupts the surface crystal lattice and introduces numerous lattice defects. These defects render N-SiC more vulnerable to laser-induced damage, and the formation of larger random cracks can adversely affect the orientation of LIPSSs (see Figure 4). Obara et al. [32] also observed random cracks induced by laser when processing N-SiC, while random cracks were not mentioned in other studies using HP-SiC [28,33]. Consequently, it is essential to monitor and control crack formation during the preparation of functional micro–nano-structures on N-SiC surfaces using ultrashort-pulse lasers. It is worth noting that the structural orientation of the LSFL is disrupted by cracks, as shown in Figure 4c, deviating from the typical perpendicular alignment relative to the polarization direction of the incident laser. Consequently, the cracks affect the orientation, density, and overall regularity of the LSFL.

### 3.2. Incident Laser Energy

**Figure 5:**
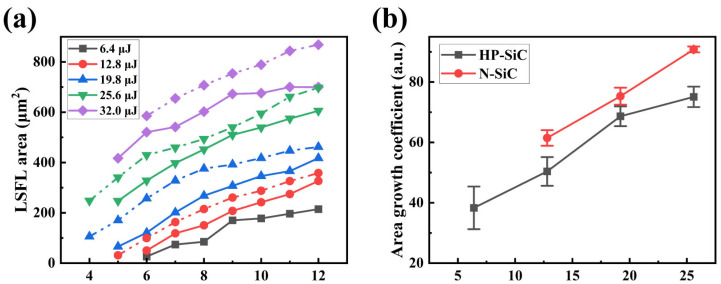
(**a**) The LSFL area produced by cumulative laser pulses at various single-pulse incident energies for different types of SiC surfaces (dashed line: N-SiC; solid line: HP-SiC); (**b**) The characteristics of the LSFL area growth coefficient as a function of incident laser energy for these SiC surfaces.

The incident laser energy is crucial for both the preparation process and the ultimate morphology of the LIPSSs. To examine the impact of single-pulse laser energy on the growth characteristics of micro–nano-structures, this study statistically analyzed the relationship between the incident laser energy and the LSFL area. Experiments were conducted using a 200 fs laser pulse duration and s-polarized incident laser, applying various single-pulse energies to fabricate LIPSSs on two types of SiC surfaces, as illustrated in Figure 5. Observations indicate that, regardless of the individual pulse energy magnitude, multiple pulses are required to form a continuous LSFL. Moreover, when subjected to the same incident laser energy, HP-SiC necessitates a greater number of pulses than N-SiC to achieve LSFL formation. Additionally, under identical energy and pulse conditions, the LSFL area on the HP-SiC surface is smaller than that on the N-SiC surface.

Meanwhile, the growth behavior of the LSFL area on SiC crystal surfaces induced by ultrashort-pulse lasers was systematically investigated. It was observed that, for both HP-SiC and N-SiC, the LSFL area increases rapidly with continued laser irradiation. During the LSFL preparation process, the LSFL area expands rapidly during the first four pulse laser irradiations, after which the rate of expansion decreases. Notably, the difference in growth rates between HP-SiC and N-SiC is observed during this initial rapid expansion phase. Consequently, our study focuses on the rapid LSFL growth in various SiC crystals during the early laser pulses. The area growth coefficient is used as a key parameter to quantify the expansion rate during LSFL formation. In our methodology, we begin counting from the first pulse that generates the LSFL structure, then record the LSFL area after three subsequent pulses, and apply linear fitting to the data. The slope of the fitted line corresponds to the LSFL area growth rate following the initial pulses at a given incident energy. The fitting equation can be expressed as follows:

$$S_{LSFL}=k*N+B$$

where $S_{LSFL}$ is the area of the LSFL, and k, N, and B represent the area growth coefficient, the number of pulsed lasers, and fitting parameter, respectively. Based on the fitting results, the area growth coefficients for HP-SiC and N-SiC under respective incident laser energy were obtained. The primary sources of uncertainty during testing arise from sample inhomogeneity and fluctuations in incident laser energy. Consequently, this study presents the results of five LSFL area growth tests conducted under identical conditions. Figure 5b illustrates the average values along with their corresponding error ranges. Furthermore, the effect of varying incident laser energies on the growth coefficients of the micro-nano-structures was statistically analyzed, as depicted in Figure 5b. The results indicate that both HP-SiC and N-SiC exhibit linear growth under increasing incident laser energy conditions. Notably, under identical incident fluence, the growth coefficient for N-SiC exceeds that of HP-SiC. The subsequent sections will provide an in-depth analysis of the underlying physical mechanisms.

### 3.3. Incident Pulse Duration

**Figure 6:**
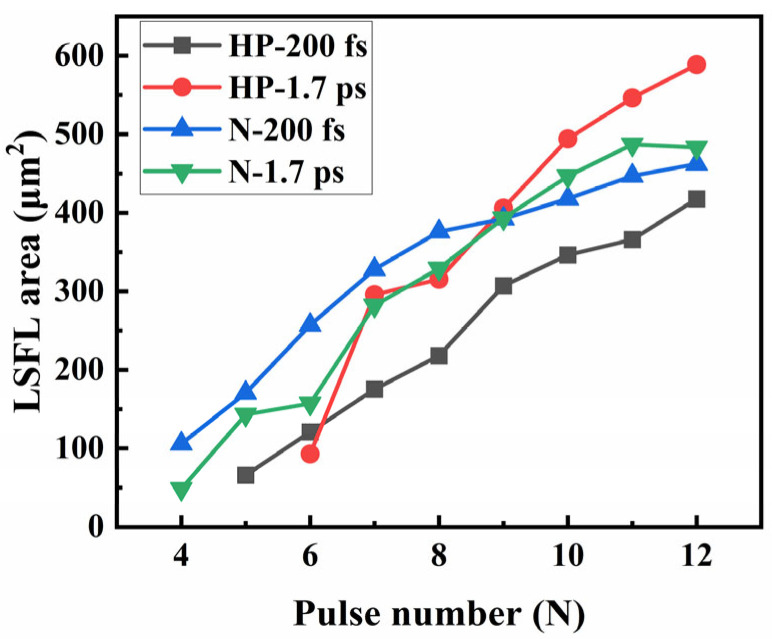
The trend of the variations in LSFL area on two types of SiC surfaces under laser irradiation with different laser duration, as well as the same laser energy (19.2 μJ) and polarization angle (90°).

The nonlinear effects induced by the pulse duration of the incident laser would significantly influence the formation of the LIPSS. In this study, four distinct pulse durations (200 fs, 600 fs, 1 ps, and 1.7 ps) were employed to irradiate different SiC surfaces. Under constant incident laser conditions—a single-pulse energy of 19.2 μJ and s-polarization—the variations in the LIPSS area were systematically investigated, thereby elucidating the impact of laser duration on the LIPSS area (as shown in Figure 6).

As illustrated by the experimental results in Figure 6, for HP-SiC, the initial LSFL area induced by 200 fs pulsed lasers exceeds that obtained using 1.7 ps lasers. Similarly, for N-SiC, fewer pulses are necessary to induce LSFLs with 200 fs pulsed lasers compared to 1.7 ps lasers. This behavior is attributed to the fact that under identical energy conditions, lasers with shorter pulse durations exhibit higher instantaneous peak power densities; meanwhile, due to the significantly larger bandgap of SiC (3.2 eV) compared to the incident laser photon energy (1.2 eV), electron transitions via multiphoton absorption are unlikely. Instead, these transitions occur through tunneling ionization, which enables electrons to overcome the barrier potential. Consequently, discrete surface ripple patterns emerge on the SiC surfaces because of this tunneling process [34,35]. This process ultimately contributes to the development of micro–nano-structures. As the laser pulses accumulate, lasers with longer pulse durations generate larger LSFL regions due to their enhanced thermal effects, which promote more effective expansion of the LSFL periphery.

### 3.4. Polarization

**Figure 7:**
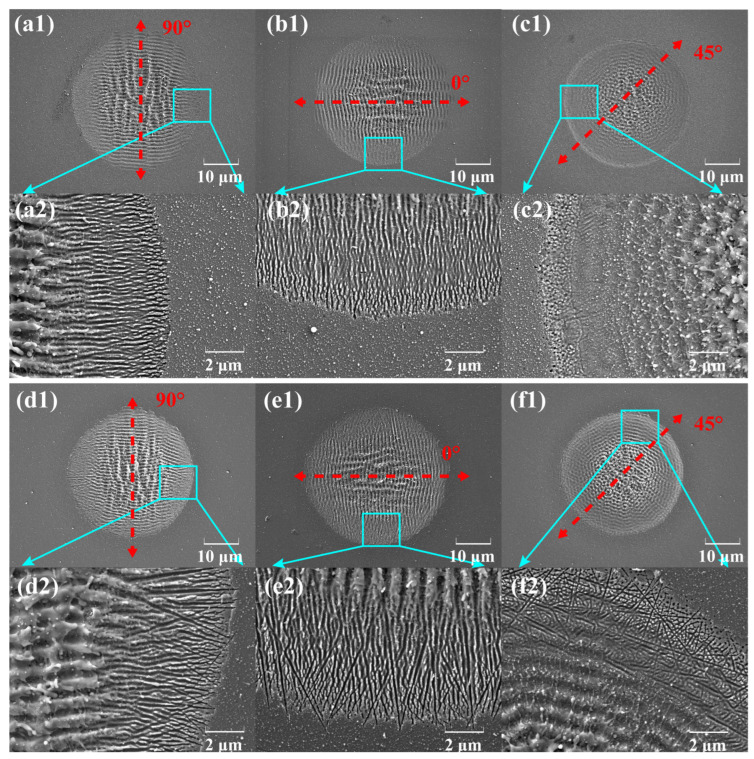
SEM images of micro–nano-structures induced by three laser polarization states on different SiC surfaces with the same incident laser parameters (pulse duration: 200 fs; incident laser energy: 19.2 μJ; pulse number: 12): (**a**–**c**) HP-SiC and (**d**–**f**) N-SiC. The images labeled with number “2” represent magnified views of the area marked in the corresponding images labeled with number “1”.

The polarization state of the incident laser would significantly influence both the direction and morphology of LIPSSs fabricated on the semiconductor surfaces using the ultrashort-pulse lasers. In this study, the LIPSSs were produced on different SiC surfaces under identical laser irradiation conductions (pulse duration: 200 fs; incident laser energy: 19.2 μJ; pulse number: 12), utilizing both polarization angles of 0° and 90° as well as intermediate polarization angles. The effects of the polarization state on the LIPSS orientation were systematically evaluated, and its influence on the LSFL area was elucidated, as shown in Figure 7.

Based on the experimental findings, incident light with polarization angles of 0° and 90° produces LSFLs and HSFLs with structural orientations that are perpendicular to the respective laser polarization directions. HSFLs are distributed in the periphery of the LIPSS area, and the closer to the center, the greater the fluctuation of HSFLs is, gradually merging into LSFLs. Unlike HP-SiC, a large number of small random cracks can be found at the edge HSFLs of N-SiC. In contrast, when the polarization angle lies between 0° and 90°, as in the case of 45°, the resulting LIPSS morphology is markedly different. Specifically, the LSFL exhibits a combined influence from both the 0° and 90° polarization components, whereas the HSFL—characterized by a high spatial frequency—is not generated under these conditions. It is worth noting that for the circular ripple structures, this experimental phenomenon was caused by splitting the initial linearly polarized light equally (90°-S polarized) using a rotating half-wave plate. This method decomposes the incident laser beam into equal components of 90° and 0° polarizations, resulting in a 1:1 ratio between the two polarization components. During the interaction of ultrashort laser pulses with the 4H-SiC surface to form ripple structures, the distinct polarization components modulated by varying electric field orientations give rise to circular ripple patterns resembling rings. Furthermore, the use of a 1:1 polarization ratio yields a circular ripple structure with uniform morphological characteristics, including consistent size and depth, ultimately resulting in a complete and homogeneous pattern. This finding indicates that future studies can tailor the polarization ratio to meet specific processing requirements, thereby achieving circular ripple structures with specific, desired attributes.

**Figure 8:**
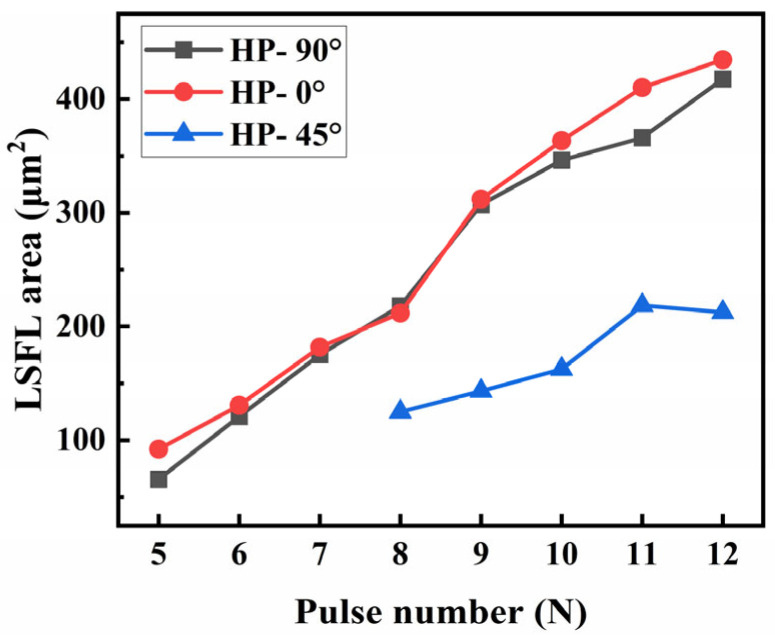
The size of the LSFL area on HP-SiC surface varies with the pulse number under the same incident laser energy (19.2 μJ), pulse duration (200 fs), and different laser polarization.

The LSFL area of HP-SiC induced by an ultrashort-pulsed laser with different polarization directions under the same incident energy conditions was statistically analyzed, as shown in Figure 8. The results indicate that the LSFL areas produced by s-polarization and p-polarization are comparable in size, whereas intermediate polarization directions lead to a notably reduced LSFL area. These findings provide an experimental basis for the fabrication of composite micro-nano structures on silicon carbide surfaces.

## 4. Discussion

As previously noted, under identical incident laser parameters during LIPSS preparation, HP-SiC and N-SiC exhibit distinct formation rates and characteristics. These differences primarily stem from variations in their material properties and the resulting micro-/nano-structures, which, in turn, influence subsequent electric field modulation. The following section explores the underlying physical mechanisms in detail.

### 4.1. Characteristics of Photothermal Weak Absorption

**Figure 9:**
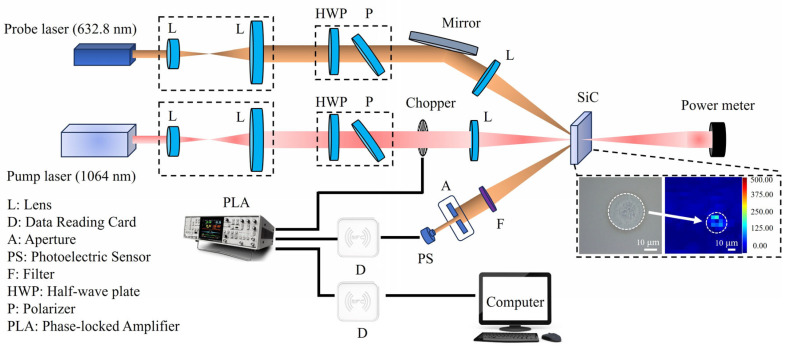
Diagram of weak photo-thermal absorption test device.

We primarily investigate the photothermal weak absorption characteristics on the laser irradiated regions of HP-SiC and N-SiC surfaces during LIPSS preparation. The photothermal weak absorption testing method is a critical technique for evaluating material absorption properties and mapping absorption defects in test samples. Its fundamental principle involves employing a high-power laser with a specific modulation frequency as the pump source, which is focused on the test sample. As the sample absorbs the pump laser energy, temperature gradients develop between the irradiated region and the adjacent nonirradiated areas, inducing thermal elastic deformation and refractive index variations—the photothermal effect. Concurrently, a low-power detection beam was irradiated at the same location, and resulting amplitude variations, influenced by the photothermal effect, were recorded to quantify the weak photothermal absorption at the test site [36,37]. In this study, the morphological structures with identical initial dimensions were first fabricated on HP-SiC (pulse duration: 200 fs; laser energy: 32 μJ) and N-SiC (pulse duration: 200 fs; laser energy: 28.8 μJ) surfaces using a single-pulse laser. Subsequently, these initial structures on both SiC surfaces were irradiated using the same laser parameters (pulse duration: 200 fs; laser energy: 19.6 μJ) for 1 to 5 pulses. A weak photothermal absorption instrument (ZC Optoelectronic Technologies Ltd., Hefei, China) was then utilized to measure the photothermal absorption values at each irradiated location, with the results presented in Figure 9. The photothermal weak absorption system consists of a pump light source and a probe light source. The pump light is generated by a quasi-continuous Nd:YAG laser (central wavelength: 1064 nm; output power: 2.5 W; repetition rate: 200 Hz) and is focused to a beam spot measuring 20 μm. The detection light is provided by a He-Ne laser operating at a central wavelength of 632.8 nm with an output power of 0.6 W. Through optical adjustments and focusing, the detection beam is configured to closely match the pump spot size and overlap with it on the surface of the test sample. By translating the test sample using a step length of 0.05 mm, the absorption characteristics across the sample’s surface are measured. When the micro-nano-structures on each SiC crystal’s surface strongly absorb the pump laser, the resulting thermal effect induces divergence in the probe beam. The corresponding photothermal weak absorption results are then derived through theoretical calculations, as illustrated in the inset in the box in Figure 9. To mitigate the effects of sample inhomogeneity and laser energy fluctuations on the test results and conclusions, five discrete locations on each of the SiC crystals were selected. Each location received different corresponding multiple laser irradiations, and the photothermal weak absorption value of each experimental position was recorded. The average value of the test results for five discrete positions was analyzed.

The experimental results demonstrate that for initial damaged pit structures with identical dimensions in HP-SiC and N-SiC, the photothermal weak absorption values at the irradiation sites in N-SiC are higher than those in HP-SiC (see Table 1). This suggests that during LIPSS preparation using ultrashort-pulse lasers, subsequent incident laser energy is more readily absorbed at the irradiated locations on the N-SiC surface than on the HP-SiC surface. Consequently, the LIPSS formation area on the N-SiC surface grows significantly faster than that on the HP-SiC surface. Moreover, this enhanced absorption is a key factor contributing to melting damage on the N-SiC surface [38]. Therefore, the variations in LIPSS fabrication characteristics resulting from differences in material absorption properties across distinct SiC surfaces are critical for achieving precise control of micro–nano-structures.

### 4.2. Electric Field Modulation Simulation

During the preparation of LSFLs on various silicon carbide substrates, we observed that under identical laser irradiation conditions, the surface ripple pattern sizes at the center of the N-SiC irradiation region were significantly larger than those at the center of the HP-SiC irradiation region. Moreover, the surface ripple pattern density on N-SiC in the direction perpendicular to the laser polarization exceeded that on HP-SiC. In contrast, in the direction of the laser polarization, both types of SiC exhibited an identical periodic surface ripple pattern distribution characteristic of LSFLs.

**Figure 10:**
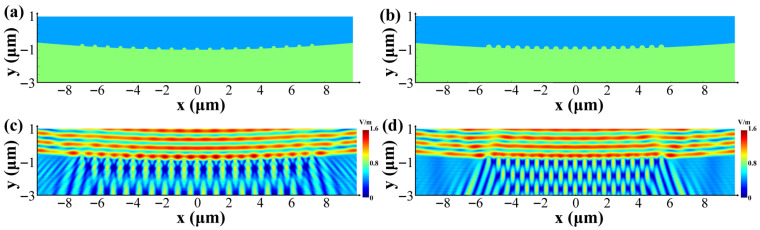
The electric field distribution on the surfaces of various SiC materials is examined under laser irradiation using identical parameters (diameter: 20 μm, amplitude: 1) on the vertical interface prior to the formation of the LIPSSz. (**a**,**c**): The structural configuration and corresponding electric field simulation outcomes for HP-SiC; (**b**,**d**): the analogous results for N-SiC.

Taking processing conditions of 200 fs, 19.2 μJ, and four pulses as an example, SEM images of the two types of SiC surfaces reveal that the average diameter of N-SiC individual fine ripples in the central region is 0.36 μm, with a vertical polarization distribution spacing of 0.6 μm. In contrast, HP-SiC individual fine ripples exhibit an average diameter of 0.26 μm and a vertical distribution spacing of 0.8 μm, while the spacing along the polarization direction is 1 μm for both materials. Based on this morphological characterization, FDTD simulations were conducted to establish micro-nano-structures that accurately represent the actual configurations observed in both materials. In the simulations presented in this paper, N-doping levels for N-SiC were maintained at 2 × 10^18^ cm^−3^, while the resistivities of HP-SiC and N-SiC were 10^10^ Ω·cm and 0.02 Ω·cm, respectively. Additionally, the wafer off-cut of each SiC crystal was set to a 4° angle along the crystal axis [0001]. These simulations analyzed the electric field modulation on surfaces modified with varying micro-nano-morphologies under the same incident laser illumination, as illustrated in Figure 10a,b. Subsequently, irradiation with an incident laser diameter of 20 μm and an amplitude of 1 was performed, as shown in Figure 10c,d. The results indicate that the larger surface ripple pattern size and higher surface ripple pattern density on the N-SiC surface induce stronger light modulation, leading to increased absorption. This enhanced absorption promotes surface ripple pattern growth until coalescence, ultimately forming the LSFLs, which consequently emerge earlier in N-SiC than in HP-SiC.

**Figure 11:**
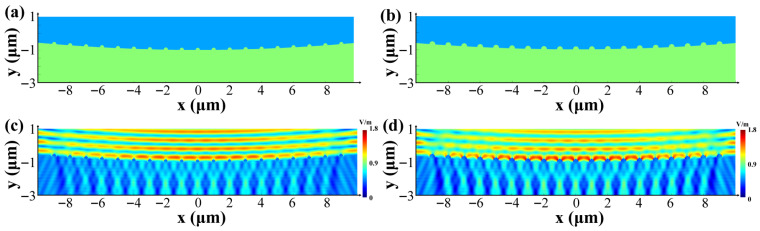
The electric field distribution on the surfaces of various SiC materials on the parallel interface prior to the formation of the LIPSSs. (**a**,**c**): HP-SiC; (**b**,**d**): N-SiC.

Meanwhile, for the case where laser polarization is parallel (as shown in Figure 11), the regions near the surface ripple patterns’ surface exhibit minimal electric field enhancement, resulting in limited laser absorption. In contrast, the areas adjacent to the surface ripple patterns’ apex demonstrate noticeable electric field amplification, thereby promoting stronger laser absorption along the sides. Moreover, N-SiC individual fine ripples tend to be slightly larger than HP-SiC individual fine ripples, which leads to marginally higher electric field modulation, increased laser absorption, and a more rapid formation of LIPSSs. Overall, the variation in electric field modulation perpendicular to the laser polarization emerges as a key factor in producing the distinct LIPSS formation processes and characteristics observed between N-SiC and HP-SiC.

In this study, it could be concluded that both the parameters of the incident laser and the properties of the sample material significantly affect the quality of LIPSS fabrication. For example, although N-SiC more readily forms LIPSSs under identical conditions, the process also induces numerous random cracks in the material. These cracks substantially compromise both the consistency and periodicity of the resulting structures, rendering them unacceptable. Consequently, stringent control over the fabrication process is imperative. Similarly, when high-pulse laser energy is employed to rapidly fabricate micro- and nano-structures under a constant laser pulse duration, central ablation phenomena are likely to occur, further necessitating precise process management.

## 5. Conclusions

This study investigates the formation characteristics and preparation mechanisms of laser-induced periodic surface structures (LIPSSs) on HP-SiC and N-SiC surfaces using ultrashort-pulse lasers with various parameters. The experimental results demonstrate that multiple ultrashort laser pulses are required to generate continuous LIPSSs on both 4H-SiC substrates. Notably, HP-SiC surfaces yield relatively regular LIPSS patterns, while N-SiC surfaces frequently exhibit random cracking, which adversely affects LIPSS quality. In addition, the study examines the impact of different single-pulse laser energies, pulse durations, and incident laser polarization states on the LIPSS formation across the two SiC surfaces. The data reveal that the LIPSS growth coefficient is linearly dependent on the single-pulse laser energy, and under equivalent energy conditions, the LIPSS area growth coefficient is higher for N-SiC than for HP-SiC. Finally, the research analyzes the roles of weak photothermal absorption and electric field modulation during the LIPSS formation process. Results indicate that, under identical laser irradiation conditions, the N-SiC surface exhibits stronger photothermal absorption and greater electric field modulation compared to HP-SiC, thereby explaining the distinct LIPSS characteristics observed between the two materials. These findings not only deepen the understanding of 4H-SiC properties but also provide a foundation for enhancing the precision processing of SiC materials.

## Figures and Tables

**Table 1:** Weak photothermal absorption of HP-SiC and N-SiC under different pulse irradiation.

Weak Photothermal Absorption (ppm)	HP-SiC (Average Value of Irradiation Position)	N-SiC (Average Value of Irradiation Position)
1th pulse	76.63	82.19
2nd pulse	82.34	88.13
3rd pulse	86.15	92.37
4th pulse	90.63	99.75
5th pulse	96.86	110.91

## Data Availability

Data underlying the results presented in this paper are not publicly available at this time but may be obtained from the authors upon reasonable request.

## Abbreviations

The following abbreviations are used in this manuscript:

SiC	Silicon carbide
LIPSS	Laser-induced periodic surface structures
HSFL	High spatial frequency LIPSS
LSFL	Low spatial frequency LIPSS
HP-SiC	High-purity 4H-SiC
N-SiC	Nitrogen-doped 4H-SiC
HWP	Half-wave plate
M	Mirror
SEM	Scanning electron microscopy
